# Supplementary material for: Extracellular Vesicles of the Plant Pathogen Botrytis cinerea
Source: J Fungi (Basel). 2023 Apr 20;9(4):495. doi: 10.3390/jof9040495 (PMC10146736; doi:10.3390/jof9040495)
Supplement: Supplementary file 1 [file jof-09-00495-s001.zip › jof-2338494-suppl-done.pdf]

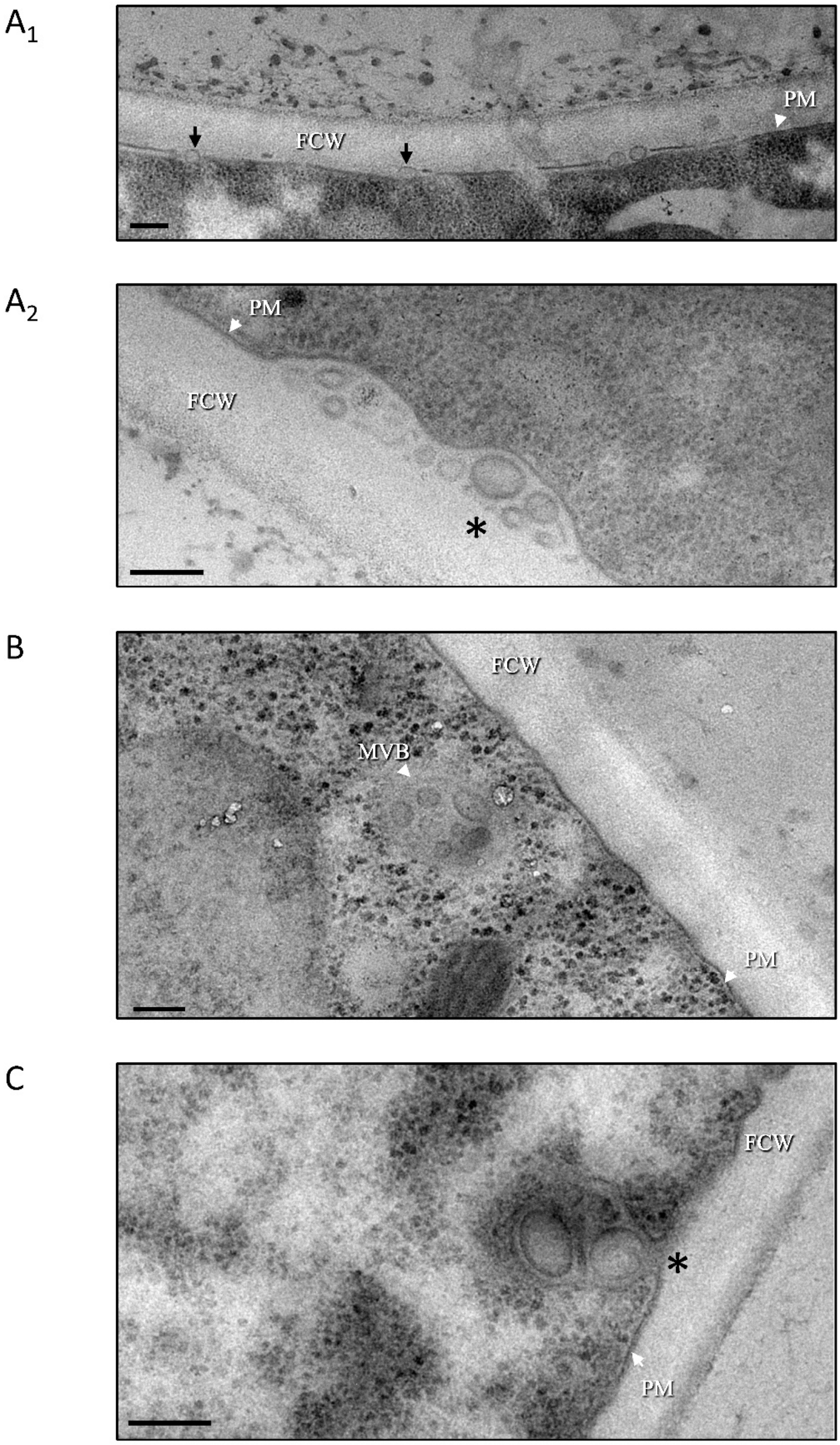

Figure S1. Hyphae of *B. cinerea* produce EVs *in vitro*. TEM of *B. cinerea* hyphae showing groups of EVs (**A1**, *) and single EVs (A2, black arrow) between the plasma membrane (PM) and the fungal cell wall (FCW), as well as multi-vesicular bodies (**B**, MVB) and likely event of MVB fusion to the PM (**C**). Scale bar: 200 nm.

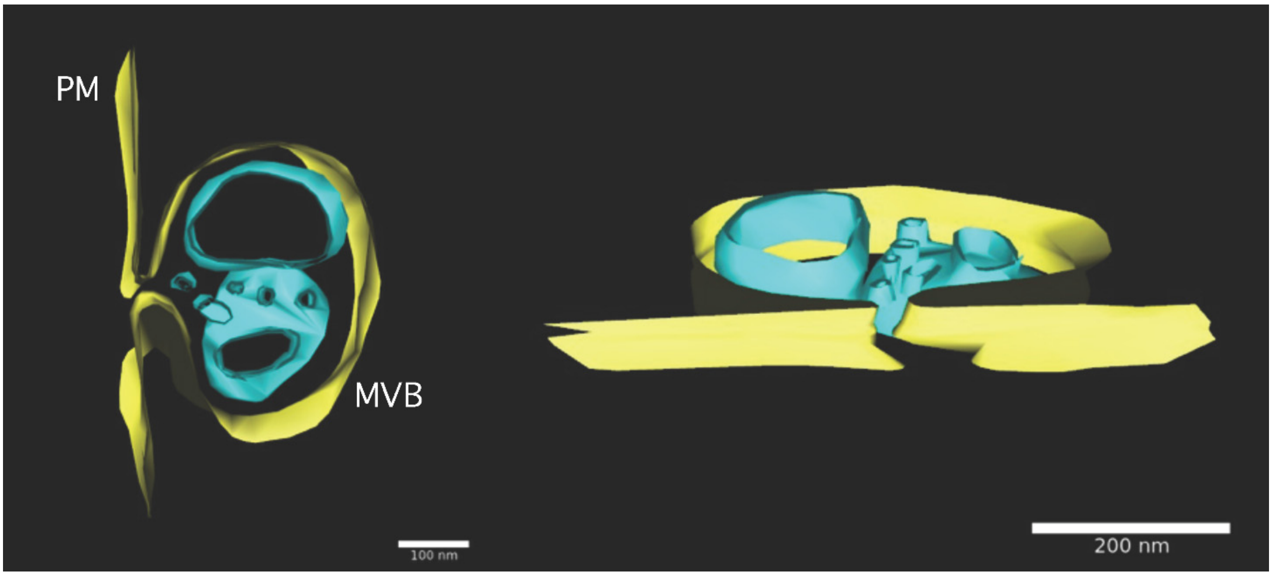

Figure S2. Electron tomography of a likely fusion event involving the plasma membrane (PM) and a MVB containing large BcEVs (blue).

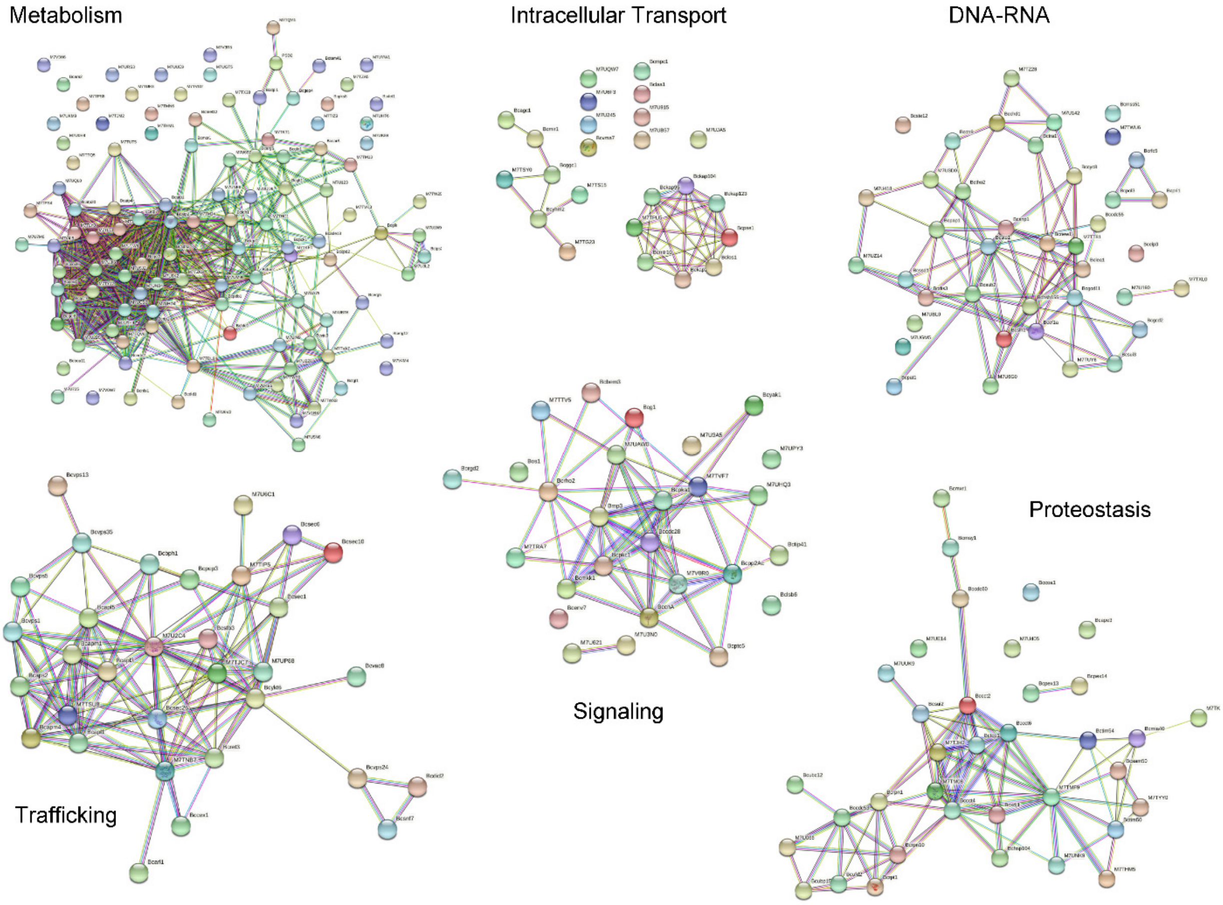

Figure S3. STRING Protein-protein interaction analyses. STRING PPI network connectivity of the BcEVs soluble proteins composing 6 of the 7 largest functional categories presented in figure 4. The metabolism network contains 113 nodes with 654 edges (vs. 81 expected edges). The Intracellular transport network contains 24 nodes with 34 edges (vs. 1 expected edges). The DNA/RNA network contains 40 nodes with 82 edges (vs. 41 expected edges). The traffic network contains 32 nodes with 122 edges (vs. 9 expected edges). The signaling network contains 27 nodes with 61 edges (vs. 7 expected edges). The proteostasis network contains 36 nodes with 84 edges (vs. 22 expected edges); Enrichment p-value < 1.0e-16 for all networks except the DNA/RNA (*p*-value = 1.36e-08). Color coding: the colored nodes in each network correspond to all the proteins classified in Table S2 under the functions Metabolism, Intracellular transport, DNA-RNA metabolism, Vesicular trafficking, Signaling or Proteostasis. Edges of purple and light blue color represent known interactions experimentally determined and from curated databases, respectively. Edges of black and yellow color represent interactions predicted from co-expression studies and text mining, respectively.

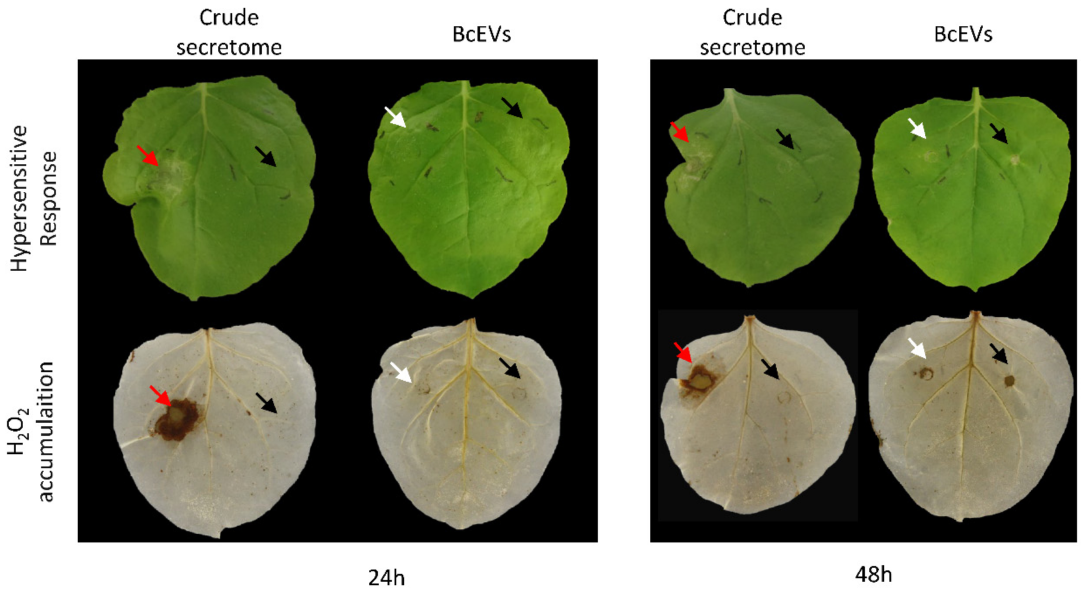

Figure S4. Toxicity of BcEVs towards tobacco leaves. *Nicotiana benthamiana* leaves (4-5weeks) were infiltrated with 100 µL of crude secretome (red arrows), BcEVs (white arrows) or PBS (Control, black arrows). The plants were incubated at 26 °C under 70% relative humidity and dark-light (17h/7h) conditions. Hypersensitive response and H_2O_2 accumulation (DAB staining) were monitored at 24h and 48h. Photographs are representative of 4 independent experiments totalizing 10 infiltrated leaves.

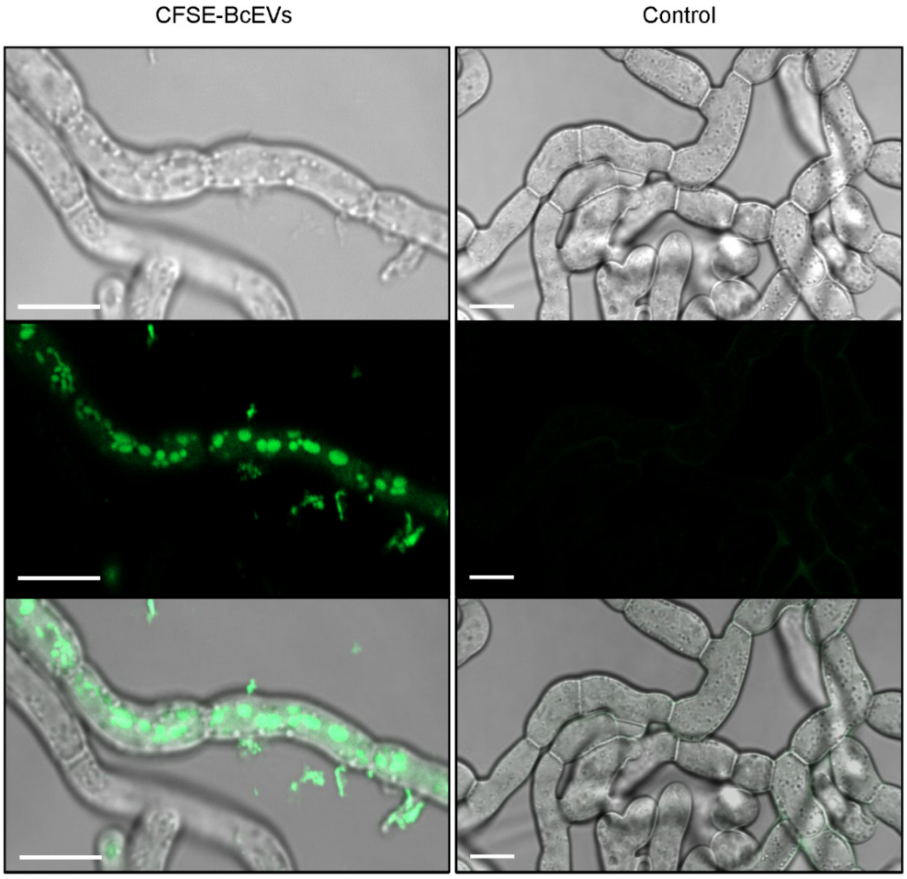

Figure S5. - Confocal microscopy of *B. cinerea* hyphae grown in the presence of CFSE-BcEVs for 55 hours. Fluorescent particles in the medium surrounding the hyphae reveal BcEVs aggregates. The absence of fluorescence was verified in the hyphae exposed to the control sample. Scale bar: 10 µm.

Table S1. List of proteins detected in the extracellular vesicles of *B. cinerea*. Proteins contained in EVs collected after fungal growth in rich solid medium (4 independent biological experiments) were analyzed by mass spectrometry. For quantification, all unique peptides of an identified protein were included, and the total cumulative abundance was calculated by summing the abundances of all peptides allocated to the respective protein. ANOVA test was applied at the protein level. The mass spectrometry proteomics data have been deposited to the ProteomeXchange Consortium via the PRIDE partner repository with the dataset identifier PXD035766.

Table S2. List of 673 proteins identified in EVs of *B. cinerea* with a maximum abundance variation of 1.5 across 4 biological replicates**.** The proposed functional classification is based on information collected as described in Material and Methods (data analysis). Clusters of predicted effectors [86] groups. Sequenceunrelated structurally similar proteins (SUSS), cluster 1 contains the MoChi1 effector of *Magnaporthe oryzae*. Cluster 8 contains the MoCDIP1 effector of *M. oryzae* and the UmRsp1 effector of *U. maydis*. Cluster 59 and 61 contain the MoPtep1 and MoFlp1 effectors of *M. oryzae*, respectively. SignalP, prediction of classical secretion signal sequence; FCW, fungal cell wall; PCWDE, plant cell wall degrading enzymes; A.A, amino acid; Enz, enzyme. ESCRT, endosomal sorting complex required for transport; ER, endoplasmic reticulum; CDIP, cell death inducing protein.
